# Optimization of MALDI Matrices and Their Preparation for the MALDI‐TOF MS Analysis of Oligonucleotides

**DOI:** 10.1002/rcm.10061

**Published:** 2025-05-07

**Authors:** Susanne Dietrich, Anja Dollinger, Andreas Wieser, Christoph Haisch

**Affiliations:** ^1^ Chair of Analytical Chemistry TUM School of Natural Sciences, Technical University of Munich Munich Germany; ^2^ Fraunhofer Institute for Translational Medicine and Pharmacology ITMP Immunology, Infection and Pandemic Research Frankfurt Germany; ^3^ Institute of Infectious Diseases and Tropical Medicine LMU University Hospital, LMU Munich Munich Germany; ^4^ German Center for Infection Research (DZIF), Partner Site Munich Germany; ^5^ Max von Pettenkofer Institute, Faculty of Medicine Ludwig‐Maximilians University Munich Munich Germany

**Keywords:** ionic matrix, MALDI‐TOF MS, mass spectrometry, oligonucleotides

## Abstract

**Rationale:**

The reproducibility of the analysis of oligonucleotides using matrix‐assisted laser desorption/ionization time‐of‐flight mass spectrometry (MALDI‐TOF MS) remains a significant challenge. This is mainly attributed to factors such as the choice and application of the matrix, as well as the inhomogeneity of the sample spots. Although previous studies have explored various parameters, such as ionic and sugar‐based matrices, and different spotting methods, none have comprehensively integrated these factors into a unified approach.

**Methods:**

Various matrices for the analysis of oligonucleotides and the effects of diverse variables, including different matrices, solvent compositions, additives, and application techniques, on the analytical performance of these matrices were investigated. Mass spectrometry analysis was conducted by a MALDI‐TOF MS in linear negative mode.

**Results:**

Our research systematically evaluates the combined effects of diverse variables to enhance the analytical performance of MALDI‐TOF MS in oligonucleotide analysis. We focused on the standard deviation of mass‐to‐charge ratios and the signal‐to‐noise (S/N) ratios. Out of 48 samples, only 19 met the S/N criteria, which is that the signals must be detectable over the whole mass range of interest (4–10 kDa).

**Conclusions:**

The ionic matrix 6‐aza‐2‐thiothymine (ATT) with 1‐methylimidazole resulted consistently in a reduced standard deviation and achieved high mass precisions. Additionally, we observed that the S/N ratios and mass precision of 3‐hydroxypicolinic acid (3‐HPA) varied significantly depending on the solvent composition and the presence of additives.

## 1 Introduction

Matrix‐assisted laser desorption/ionization time‐of‐flight mass spectrometry (MALDI‐TOF MS) is a highly effective analytical technique, particularly renowned for its application in protein analysis. Its key strengths include the ability to ionize large, complex molecules without significant fragmentation, leading to accurate mass determination and biomolecule identification. MALDI‐TOF MS also offers several practical advantages, such as simplicity, speed, low reagent and sample consumption, and straightforward mass spectrum interpretation [1].

Despite its success in protein analysis, MALDI‐TOF MS is less popular for the analysis of oligonucleotides, which are short DNA and RNA segments. Oligonucleotides are small molecules and are more prone to fragmentation under MALDI conditions than larger biomolecules such as proteins. This often results in complex spectra with overlapping signals, complicating the data interpretation. Additionally, the ionization efficiency of oligonucleotides in MALDI‐TOF MS is generally lower compared to proteins, making the analysis, and accurate mass determination more challenging [2, 3, 4, 5, 6, 7, 8, 9].

One of the major challenges in oligonucleotide investigation using MALDI‐TOF MS is the formation of alkali metal adducts. These adducts, such as phosphodiester salts with Na^+^ or K^+^, can significantly affect mass resolution and detection limits. The signal corresponding to the intact oligonucleotide is often observed as a distribution of peaks at higher m/z values, rather than as a single molecular ion peak, further complicating the analysis [10, 11].

Oligonucleotides play crucial roles in various biological processes. For example, microRNAs (miRNAs), short non‐coding RNAs ranging from 19 to 23 nucleotides in length, are involved in gene regulation by targeting specific messenger RNAs, leading to their degradation or inhibition of translation [12]. Given their biological importance, oligonucleotides are valuable biomarkers in genomics, molecular diagnostics, and drug development, facilitating early disease detection and personalized medicine approaches [13, 14].

To overcome the limitations in oligonucleotide analysis, one promising approach is the optimization of the matrix used in MALDI‐TOF MS. By improving the chemical composition and properties of the matrix, it is possible to enhance the ionization efficiency and stability of oligonucleotides during the MALDI process. Optimized matrices can also reduce metal adduct formation and fragmentation, leading to more consistent and accurate mass spectra [15]. These advancements are essential for fully exploiting the potential of MALDI‐TOF MS in oligonucleotide analysis, ultimately aiding in developing more reliable and effective diagnostic and therapeutic tools.

Research regarding the optimization of MALDI‐TOF MS matrices was already done by Pieles et al., who reported that diammonium hydrogen citrate (DAC) as an additive in 2,4,6‐trihydroxyacetophenone (THAP) caused suppression of alkali ion adducts. Additionally, it seems that the additive plays an important role in enhancing the desorption/ionization of the intact oligonucleotides. Peak widening was prevented, which resulted in an increase in signal intensity and resolution [8]. Also, Distler and Allsion showed that the addition of fucose to 6‐aza‐2‐thiothymine (ATT) or 3‐hydroxypicolinic acid (3‐HPA) led to a reduction of fragmentation and an increase in spot homogeneity and signal intensity [16]. With this publication, it becomes apparent that also simple additives such as monosaccharides can influence the quality of oligonucleotide analysis. Beneath conventional matrices, Armstrong et al. mentioned ionic liquid matrices [17], which were later described by the more general term ionic matrices (IMs) [18]. In the recent years, they have been applied in the analysis of various biomolecules, including oligonucleotides. Typically, IMs are solid or liquid organic salts generated by mixing conventional matrices (e.g., THAP, ATT, or 3‐HPA) with an organic base (e.g., pyridine, 1‐methylimidazole, or butylamine) in equimolar amounts. Samples prepared with these matrices form less non‐homogeneous spots and feature improved mass spectrum reproducibility and enhanced sensitivity [17, 18, 19, 20, 21, 22, 23].

The existing literature presents different advancements in optimizing matrix formulations. However, these distinct approaches have not yet been integrated into a comprehensive framework. In this study, we systematically evaluate the combined effects of various factors, including different matrices, solvent compositions, additives, and application techniques, to enhance the analytical performance of MALDI‐TOF MS in oligonucleotide analysis. Our goal is to address the current challenges and improve the reliability and accuracy of this technique.

## 2 Experimental

### 2.1 Chemicals and Reagents

The Oligonucleotide Calibration Standard was purchased from Bruker (Bruker Daltonik GmbH, Bremen, Germany). ATT (98%), D‐arabinose (≥ 99.0%), pyridine (≥ 99.0%), tributylamine (≥ 98.5%), and 1‐methylimidazole (1‐MI) (99%) were supplied by Thermo Fisher Scientific (Schwerte, Germany). 3‐HPA (≥ 99.0%), 2,5‐dihydroxybenzoic acid (2,5‐DHBA) (≥ 99.0%), sinapinic acid (SA) (≥ 98%), 2,4,6‐THAP (≥ 99.5%), α‐cyano‐4‐hydroxycinnamic acid (HCCA) (≥ 99.0%), DAC (≥ 99.0%), acetonitrile (ACN) (≥ 99.9%), butylamine (99.5%), and D‐glucose (≥ 99.5%) were from Sigma‐Aldrich (Taufkirchen, Germany). D‐fructose was obtained by Merck KGaA (Darmstadt, Germany). Ethylenediaminetetraacetic acid disodium salt dihydrate (EDTA) (99%) and the solvents 2‐propanol (≥ 99.5%), ethanol (EtOH), and methanol (MeOH) were purchased from Carl Roth GmbH – Co. KG (Karlsruhe, Germany). All solvents and reagents were at least analytical reagent grade.

### 2.2 Sample Preparation

#### 2.2.1 Solvent Mixtures

In addition to testing different matrix salts (3‐HPA, ATT, 2,5‐DHBA, SA, HCCA, and 2,4,6‐THAP), four different solvent mixtures were tested: ACN/H_2_O (1:1, vol/vol), 2‐propanol/H_2_O (1:1, vol/vol), ACN/2‐propanol/H_2_O (1:1:1, vol/vol), and ACN/EtOH/H_2_O (1:1:1, vol/vol). All solvent mixtures were stored at 4 °C.

#### 2.2.2 Additives

DAC and EDTA were dissolved at a concentration of 10 mg mL^−1^. While EDTA was only tested in ACN/H_2_O, DAC was dissolved in all solvent mixtures.

D‐fructose, D‐glucose, and D‐arabinose were dissolved in the DAC solution at a concentration of 5 mg mL^−1^. D‐glucose and D‐arabinose were only tested in the ACN/H_2_O DAC solution, whereas D‐fructose was additionally tested in three other DAC solvent mixtures (2‐propanol/H_2_O, ACN/2‐propanol/H_2_O, and ACN/EtOH/H_2_O).

#### 2.2.3 Conventional Matrices

3‐HPA, ATT, 2,5‐DHBA, SA, HCCA, and 2,4,6‐THAP were prepared as saturated solution in ACN/H_2_O with DAC and EDTA as additive, respectively. 3‐HPA was also tested with all other additive solutions. All matrices were prepared at room temperature with freshly prepared additive solutions.

#### 2.2.4 Ionic Matrices

Organic salts were prepared according to the method described by S. Carda‐Brock et al. with slight modifications [18]. ATT, 3‐HPA, and 2,4,6‐THAP were dissolved in MeOH (20 mg mL‐1) with an equimolar amount of pyridine, butylamine, tributylamine, and 1‐methylimidazole (1‐MI), respectively. The mixture was vortexed for 5 min and evaporated to dryness. The dried organic salt was dissolved in the respective additive solution (75 mg mL^−1^).

#### 2.2.5 Spotting Method

The matrices were tested with common sample deposition approaches, i.e., the *dried droplet method* and *two‐layer method*s. For *dried droplet*, the matrix solution is mixed with the liquid sample and spotted (1 μL) onto the MALDI target plate (Mix). In the *two‐layer* approaches, a distinction is made between the order of spotting matrix and sample. Either first 0.5 μL matrix solution is deposited on the target and is allowed to dry, followed by depositing 0.5 μL oligo sample (M‐O) on the same spot, or vice versa (O‐M).

### 2.3 MALDI‐TOF MS Analysis

A polished ground steel MALDI target plate (Bruker Daltonik GmbH, Bremen, Germany) was used for MALDI‐TOF MS measurements, which were performed with an Autoflex Speed mass spectrometer (Bruker Daltonik GmbH, Bremen, Germany). The MS is equipped with a 1 kHz smartbeam‐II laser and controlled by the FlexControl software (version: 3.4.135.7, Bruker Daltonik GmbH, Bremen, Germany). Spectra were recorded in negative linear mode in the mass range between 2000 and 15 000 Da. The parameter settings were optimized as follows: ion source 1:19.50 kV, ion source 2:18.35 kV, pulsed ion extraction time: 330 ns. Gain and laser power were set to the manufacturer's recommended values for detecting oligonucleotides.

An external standard was used for instrument calibration and as reference sample to compare different matrices, containing three oligonucleotides (12‐, 20‐, and 30‐mer) with masses between 4000 and 10 000 Da (Oligonucleotide Calibration Standard, Bruker Daltonik GmbH, Bremen, Germany). All spectra are based on the average of 5000 pulses, accumulated from different points of the target. Data processing was performed using the FlexAnalysis software (version: 3.4.76, Bruker Daltonik GmbH, Bremen, Germany).

### 2.4 Microscope Imaging

Microscopic images were recorded by a WITec alpha 300A Raman microscope (WITec GmbH, Ulm, Germany) with a 50× objective (Zeiss LD Epiplan NEOFLUAR 50× 0.55 from Carl Zeiss AG, Germany).

### 2.5 Data Processing and Statistical Analysis

Parameters compared were calculated using the singly charged molecular ions in negative ion mode ([M‐H]^−^) of the three oligonucleotides (12‐, 20‐, and 30‐mer) with theoretical m/z‐values of 3646.4 Da (Oligo 12), 6118.0 Da (Oligo 20), and 9192.0 Da (Oligo 30). The mass accuracies are displayed in ppm.

The signal‐to‐noise (S/N) ratio was determined using the build‐in function of the program FlexAnalysis. This function calculates the ratio of a specific peak to the standard deviation of the background on both sides of the peak, where no significant signal is detected (noise). The S/N ratio is then given as peak height to three times this standard deviation. This value is considered a reasonable estimate for the limit of detection [24]. We consider S/N ratio values above 1 as threshold for significant peaks.

All data calculations were performed using Origin (OriginPro 2023b) and Microsoft Excel (Microsoft Office Professional Plus 2021), plots were generated employing R Studio (2024.04.2).

## 3 Results and Discussion

Six common matrix materials (3‐HPA, ATT, 2,5‐DHBA, SA, 2,4,6‐THAP, and HCCA) were tested, as well as 12 different ionic matrix combinations, different solvent mixtures, and different additives, such as DAC, EDTA, and the sugars D‐fructose, D‐glucose, and Darabinose. An overview of all tested components can be seen in Table 1.

**TABLE 1:** Tested matrices with their structure, the used organic base in case of ionic matrices, the tested additives, and solvent mixtures.

Matrix	Structure	Org. base	Additive	Solvent
HCCA	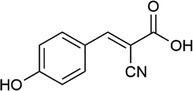		DAC	ACN/H_2_O
			EDTA	
SA	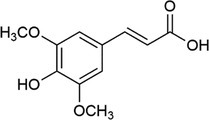		DAC	ACN/H_2_O
			EDTA	
2,5‐DHBA	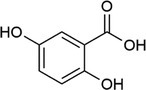		DAC	ACN/H_2_O
			EDTA	
2,4,6‐THAP	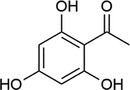		DAC	ACN/H_2_O
			EDTA	
2,4,6‐THAP (IM)		Pyridine	DAC	ACN/H_2_O
		Butylamine		
		Tributylamine		
		1‐MI		
3‐HPA	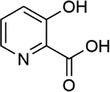		DAC resp. DAC, D‐Fru	ACN/H_2_O
				2‐Propanol/H_2_O
				ACN/2‐propanol/H_2_O
				ACN/EtOH/H_2_O
			DAC, D‐Glu	ACN/H_2_O
				ACN/2‐propanol/H_2_O
			DAC, D‐Ara	ACN/H_2_O
				ACN/2‐propanol/H_2_O
3‐HPA (IM)		Pyridine	DAC	ACN/H_2_O
		Butylamine		
		Tributylamine		
		1‐MI		
ATT	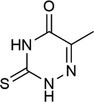		DAC	ACN/H_2_O
			EDTA	
ATT (IM)		Pyridine	DAC resp. DAC, D‐Fru	ACN/H_2_O
		Butylamine		2‐Propanol/H_2_O
		Tributylamine		ACN/2‐propanol/H_2_O
		1‐MI		ACN/EtOH/H_2_O

We identified two critical parameters for evaluating the performance of a MALDI matrix. Signals must be detectable over the whole mass range of interest (4–10 kDa). Matrices with one of the three signals below a threshold value of 1 were excluded for further investigation. Thus, we only considered the detection of all three signals, while the quantitative signal intensity is not considered, as MALDI is not routinely used for quantitative analyses. The second parameter we evaluated is the precision in determining the mass, which is represented by the standard deviation of the found m/z values. The accuracy for the mass determination, i.e. the difference between the measured m/z values and the correct mass, was neglected as the ability to correctly determine the absolute mass of a sample by MALDI depends on the mass calibration of the instrument for specific measuring conditions.

Hence, all matrices resp. additives were checked for the presence of signals for the three reference samples, as well as the standard deviation of the detected masses for these three samples. This statistic evaluation was calculated from 10 repetitive measurements (*n* = 10).

### 3.1 Conventional Matrices/Additives

Four of the six tested matrices, even when combined with the additives EDTA or DAC, did not meet the criteria for successful detection, as they failed to produce significant signals for each of the three oligonucleotide masses (S/N). As an additive, EDTA provided no improvement in signal quality across any of the matrices. In contrast, DAC consistently enhanced signal intensity for all matrices, although only 3‐HPA produced signals for all three oligonucleotide masses with a S/N above the predefined threshold. This underscores the crucial role of DAC in boosting ionization while emphasizing that the choice of matrix is pivotal in achieving successful and reliable detection.

**FIGURE 1:**
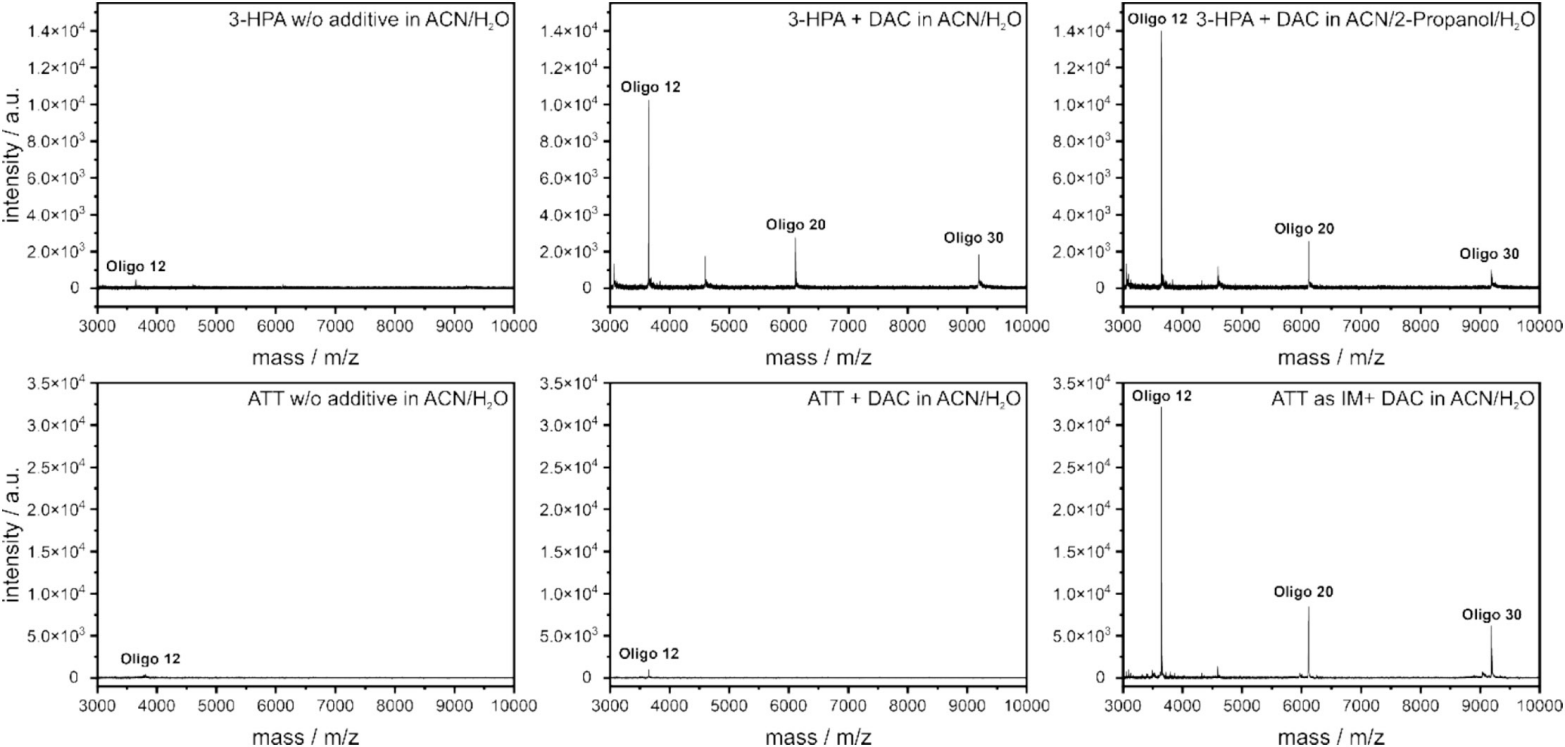
Exemplary spectra of (A) 3‐HPA in ACN/H_2_O without additive (left), with DAC as additive (middle), and with DAC in the solvent mixture ACN/2‐propanol/H_2_O (right). (B) ATT in ACN/H_2_O without additive (left), with DAC as an additive (middle), and as an ionic matrix (with 1‐methylimidazole as an organic base) with DAC (right). The unlabeled signals in the spectra are z = 2 ions. For each measurement, 0.5 μL sample and 0.5 μL matrix solution were investigated by 5000 laser pulses.

Distinct differences were observed between 3‐HPA and ATT. For both matrices, the absence of additives resulted in the poorest performance, yielding lower S/N ratios. As already mentioned, adding specific additives such as DAC improves signal quality, as shown in the left and middle spectra of Figure 1. When using only the pure matrix without additive, only Oligo 12 was detected, though with low intensity. With the ATT matrix, no additional signal could be detected, even after adding DAC. However, DAC increased the signal detected for Oligo 12. 3‐HPA had a different effect: after adding DAC to the matrix, not only higher signal intensities were detected, but also signals for Oligo 20 and 30 appeared.

Earlier studies suggest that monosaccharides such as fucose improve the performance of a MALDI matrix [16, 25]. In addition to D‐fructose, two other monosaccharides (D‐glucose and D‐arabinose) were tested in combination with DAC. The addition of D‐fructose resulted in an improvement in the S/N values. In contrast, the use of D‐arabinose resulted in an inconsistent detection of oligonucleotides. Adding D‐glucose led to lower signal intensities compared to conditions without the monosaccharide.

### 3.2 Solvents

Another investigated parameter was the solvent composition, which was tested on 3‐HPA, and ATT. In addition to the standard solvent mixture of ACN and H_2_O, three other mixtures were investigated: 2‐propanol/H_2_O, ACN/2‐propanol/H_2_O, and ACN/EtOH/H_2_O. As shown in Figure 1 (row A, right spectrum), further S/N optimization could be observed for 3‐HPA when combined with DAC and ACN/2‐propanol/H_2_O as a solvent mixture. It seems that some solvent mixtures can have a positive influence on the measurements of oligonucleotides. All four solvent mixtures had either a positive or no significant impact and were used for further investigations.

### 3.3 Ionic Matrices (IMs)

Beyond the evaluation of the conventional matrices, three matrices (ATT, 3‐HPA, and 2,4,6‐THAP) were investigated as exampled for ionic matrices, combined with four organic bases (pyridine, butylamine, tributylamine, and 1‐MI). No IM fulfilled our criteria, but ATT with 1‐MI as organic base provided the highest S/N ratios of all matrices, demonstrating superior ionization properties (Figure 1b, right).

While results so far suggest that 3HPA and ATT are not effective alone, their performance is significantly enhanced in the presence of additives. In the following, 3‐HPA and ATT as IM‐ were systematically investigated with two additive combinations: with DAC as an additive and with DAC in combination with D‐fructose. All four solvent mixtures were used so that a total of 16 samples was analyzed.

**FIGURE 2:**
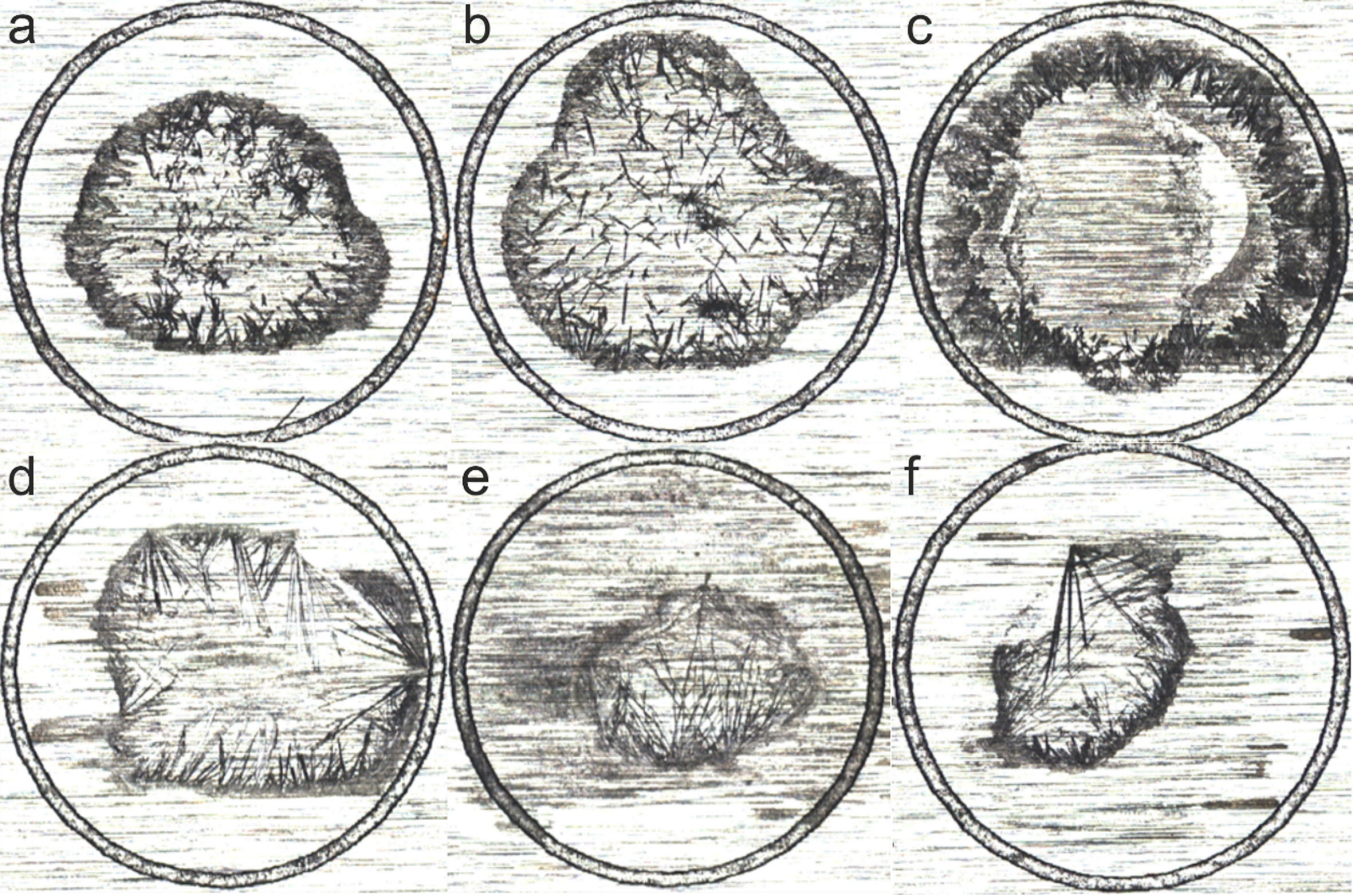
Microscopic images with a 50× magnification of six different matrix spots (1 μL) on a ground steel target. (a) 1‐MI ATT ACN EtOH H_2_O DAC (Mix), (b) 1‐MI ATT ACN H_2_O DAC fructose (Mix), (c) 1‐MI ATT ACN H_2_O DAC (M‐O), (d) 3‐HPA ACN H_2_O DAC (O‐M), (e) 3‐HPA ACN EtOH H_2_O DAC fructose (O‐M), (f) 3‐HPA ACN H_2_O DAC fructose (O‐M). The circle marks the MALDI spot on the target.

The various matrices, each with different additives and solvent compositions, exhibited highly distinct crystallization patterns, but all with a characteristic coffee stain. This is exemplified in Figure 2, which presents microscope images of three 3‐HPA matrices (upper row) and three ionic ATT matrices (bottom row)—highlighting the most pronounced optical differences in crystallization.

**FIGURE 3:**
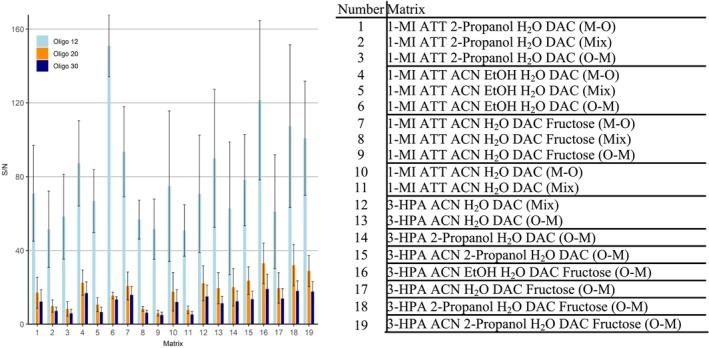
Mean S/N values of the three evaluated oligonucleotides for ATT and 3‐HPA, applied with different spotting orders (Matrix‐Oligo M‐O, Mix, and Oligo‐Matrix, O‐M), different solvent combinations, and additives. Error bars represent one standard dev. with *n* = 10.

All samples were tested with different spotting orders to assess any potential effects on the outcomes. Figure 3 displays each “1‐MI ATT” and “3‐HPA” sample, along with the respective spotting order, that met the criteria of generating signals for all three oligonucleotide masses with the required S/N ratio. Out of the total 48 samples, only 19 fulfilled these criteria. Remarkably, the spotting order had a significant impact on reproducibility. The O‐M order was effective in 10 of the 19 successful samples, while the M‐O order worked for only four, and the Mix approach for five samples.

Oligo 12 consistently displayed the highest S/N ratio, likely due to the inverse relationship between molecular weight and ionization efficiency, where smaller oligonucleotides ionize more readily and produce stronger signals than larger ones. Oligo 12 exceeds a S/N of 50 in all samples, with the highest values over 100 observed in samples 1‐MI ATT ACN EtOH H_2_O DAC (O‐M) (**6**), 3‐HPA ACN EtOH H_2_O DAC fructose (O‐M) (**16**), 3‐HPA 2‐propanol H_2_O DAC fructose (O‐M) (**18**), and 3‐HPA ACN 2‐propanol H_2_O DAC fructose (O‐M) (**19**). For Oligo 20 and Oligo 30, similar S/N ratios were observed across these samples (S/N_Oligo 20_ = 28.8–33.0; S/N_Oligo 30_ = 5.0–19.1).

**FIGURE 4:**
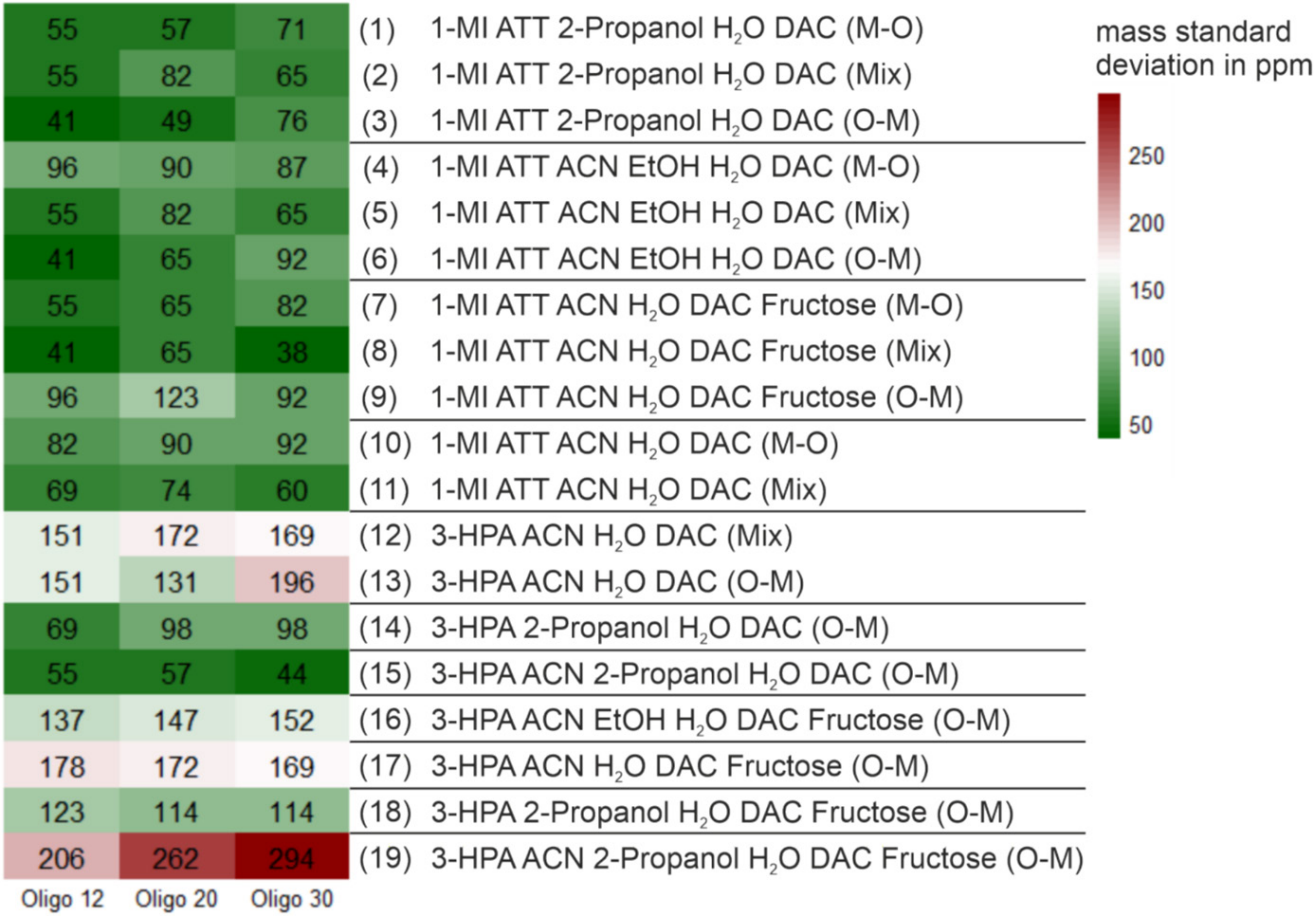
Heatmap of the mass precision in ppm of Oligo 12, 20, and 30 signals of the tested matrices ATT and 3‐HPA with different spotting orders (Matrix‐Oligo; M‐O, Mix, or Oligo‐Matrix; O‐M), different solvent combinations, and additives. The standard deviations range between 38 ppm (dark green) and 294 ppm (dark red).

For all 19 samples, the standard deviation of the m/z values of 10 measurements were calculated, as displayed in Figure 4.

Most samples showed deviations in a range of 38 to 150 ppm, with only 3‐HPA ACN H_2_O DAC (Mix) (**12**), 3‐HPA ACN H_2_O DAC (O‐M) (**13**), 3‐HPA ACN EtOH H_2_O DAC fructose (O‐M) (**16**), 3‐HPA ACN H_2_O DAC fructose (O‐M) (**17**), and 3‐HPA ACN 2‐propanol H_2_O DAC fructose (O‐M) (**19**) exceeding this range. The spectra of 3‐HPA ACN H_2_O DAC fructose (O‐M) (**17**) and 1‐MI ATT ACN EtOH H_2_O DAC (O‐M) (**6**), shown in the Supporting Information (S1), are provided for comparison.

It is noteworthy that the composition 3‐HPA ACN 2‐propanol H_2_O DAC fructose (O‐M) demonstrated the best performance, exhibiting high signal‐to‐noise (S/N) ratios. Similarly, the mixture 3‐HPA ACN EtOH H_2_O DAC fructose (O‐M) also showed strong performance in this regard. However, both compositions yield a low mass precision.

In contrast, almost all 1‐MI ATT mixtures (samples **1–11**) showed low mass deviations (38–123 ppm). This result is remarkable as this specific combination of matrix and organic base had not been tested previously. The findings highlight its potential as a novel approach for improving ionization efficiency, and precise m/z detection.

## 4 Conclusions

For the first time, the combined effects of various additives, different solvent mixtures on six standard matrices, and 12 ionic matrices were tested for MALDI‐TOF MS analysis of oligonucleotides. Among the standard matrices, only 3‐HPA met the criteria, demonstrating a notable enhancement in signal quality for all tested samples when supplemented with additives such as DAC or D‐fructose. Also, the selection of the optimum solvent composition showed a positive impact on the S/N ratio.

Regarding ionic matrices, none fulfilled the criteria, but the combination of ATT with 1‐MI as an organic base, described for the first time, provided high S/N ratios and low standard deviations of the mass analysis, demonstrating superior ionization and high mass accuracy. The investigation further confirms that the spotting order significantly impacts reproducibility. Out of 48 samples, only 19 met the S/N criteria, with 1‐MI ATT mixtures and 3‐HPA 2‐propanol H_2_O DAC fructose (O‐M) achieving the best results with consistently high S/N ratios and high mass precisions.

To our best knowledge, no further study has investigated a combined approach of additive, solvent mixture, and ionic matrix. Our research showed significant differences in signal quality and reproducibility depending on the matrix combination used. There may be other matrices featuring even better performance for oligonucleotide analysis, as the respective ionization mechanisms are not yet fully understood. This area of research remains open, highlighting the potential for discovering new or optimized matrix systems with superior analytical properties.

## Author Contributions

**Susanne Dietrich˟:** conceptualization, methodology, investigation, visualization. **Anja Dollinger:** conceptualization, investigation, methodology, writing – original draft, visualization. **Andreas Wieser:** writing – review and editing, project administration, methodology, formal analysis. **Christoph Haisch:** methodology, validation, writing – review and editing, project administration, conceptualization, supervision.

### Peer Review

The peer review history for this article is available at https://www.webofscience.com/api/gateway/wos/peer‐review/10.1002/rcm.10061.

## Supporting information


**Figure S1** Mass spectra of three technical replicates (a,b,c) of the MALDI Matrix 1‐Methylimidazole (1‐MI) with 6‐aza‐2‐thiothymine (ATT) in ACN, EtOH, H_2_O, and DAC (sample 6). The oligo‐standard (Oligo 12, 20, 30) was spotted prior to the Matrix (O‐M). Adducts are highlighted with a red dot.
Figure S2 Mass spectra of three technical replicates (a,b,c) of the MALDI Matrix in ACN, H_2_O, DAC, and fructose (sample 17). The oligo‐standard (Oligo 12, 20, 30) was spotted prior to the Matrix (O‐M). Adducts are highlighted with a red dot.

## Data Availability

The data that support the findings of this study are available from the corresponding author upon reasonable request.
