# Supplementary material for: Optimization of MALDI Matrices and Their Preparation for the MALDI‐TOF MS Analysis of Oligonucleotides
Source: Rapid Commun Mass Spectrom. 2025 May 7;39(15):e10061. doi: 10.1002/rcm.10061 (PMC12059521; doi:10.1002/rcm.10061)
Supplement: Supplementary file 1 — Figure S1 Mass spectra of three technical replicates (a,b,c) of the MALDI Matrix 1‐Methylimidazole (1‐MI) with 6‐aza‐2‐thiothymine (ATT) in ACN, EtOH, H2O, and DAC (sample 6). The oligo‐standard (Oligo 12, 20, 30) was spotted prior to the Matrix (O‐M). Adducts are highlighted with a red dot. Figure S2 Mass spectra of three technical replicates (a,b,c) of the MALDI Matrix in ACN, H2O, DAC, and fructose (sample 17). The oligo‐standard (Oligo 12, 20, 30) was spotted prior to the Matrix (O‐M). Adducts are highlighted with a red dot. [file RCM-39-e10061-s001.pdf]

Supporting Information

Optimization of MALDI Matrices and their Preparation for the MALDI-TOF MS Analysis of Oligonucleotides

Susanne Dietrich[×1,2], Anja Dollinger[×1,3], Andreas Wieser[2,3,4,5], Christoph Haisch[1,2]

1: Chair of Analytical Chemistry, TUM School of Natural Sciences, Technical University of Munich, Munich, Germany

2: Fraunhofer Institute for Translational Medicine and Pharmacology ITMP, Immunology, Infection and Pandemic Research, Germany

3: Institute of Infectious Diseases and Tropical Medicine, LMU University Hospital, LMU Munich Germany

4: German Center for Infection Research (DZIF), Partner Site, Munich, Germany

5: Max von Pettenkofer Institute, Faculty of Medicine, Ludwig-Maximilians University Munich, Germany

[×] shared first authors

Corresponding author:
Christoph Haisch
Lichtenbergstraße 4
85748 Garching
Germany
Haisch@tum.de
+49 89 289-154 504

1 SI Comparison of Representative MALDI Spectra for the Matrices (6) and (17)

Two MALDI matrices were selected based on their ability to generate signals for all three target oligonucleotide masses with the required signal-to-noise (S/N) ratio. Out of 48 tested samples, only 19 met this criterion, including compounds 1-methylimidazole (1-MI) with 6-aza-2-thiothymine (ATT) in ACN, EtOH, H_2O, and DAC (**6**) and 3-hydroxypicolinic acid (3-HPA) in ACN, H_2O, DAC, and Fructose (O-M) (**17**), which are presented exemplary (Figures S1, and S2). For a quantitative evaluation of all tested samples, please refer to the main manuscript.

The MALDI-TOF spectra for compound (**6**) display consistent signal intensities across the three technical replicates, with strong and reproducible peaks observed for all three oligonucleotides. In contrast, compound (**17**) showed markedly higher variability between replicates and generally lower intensities, particularly for the higher mass oligonucleotides. The mass spectrum of compound (**6**) displays a series of additional signals that are consistent with, and may be attributed to, adduct formation with sodium (+22 Da) and potassium (+38 Da). These presumed adduct peaks are indicated by red markers in the spectrum. Compound (**17**) displays a pattern of signals that may likewise be attributed to adduct formation with sodium and potassium. In contrast to compound (**6**), the putative alkali metal adduct peaks are more pronounced and exhibit higher relative intensities compared to the corresponding oligonucleotide signals, suggesting a potentially stronger interaction with alkali metal ions. Furthermore, replicate variability is notably increased for compound (17), particularly in the mid- to high-mass regions of the spectrum. Overall, compound (**6**) provided more robust and reproducible spectra with lower adduct interference, supporting its superior performance under the tested conditions.

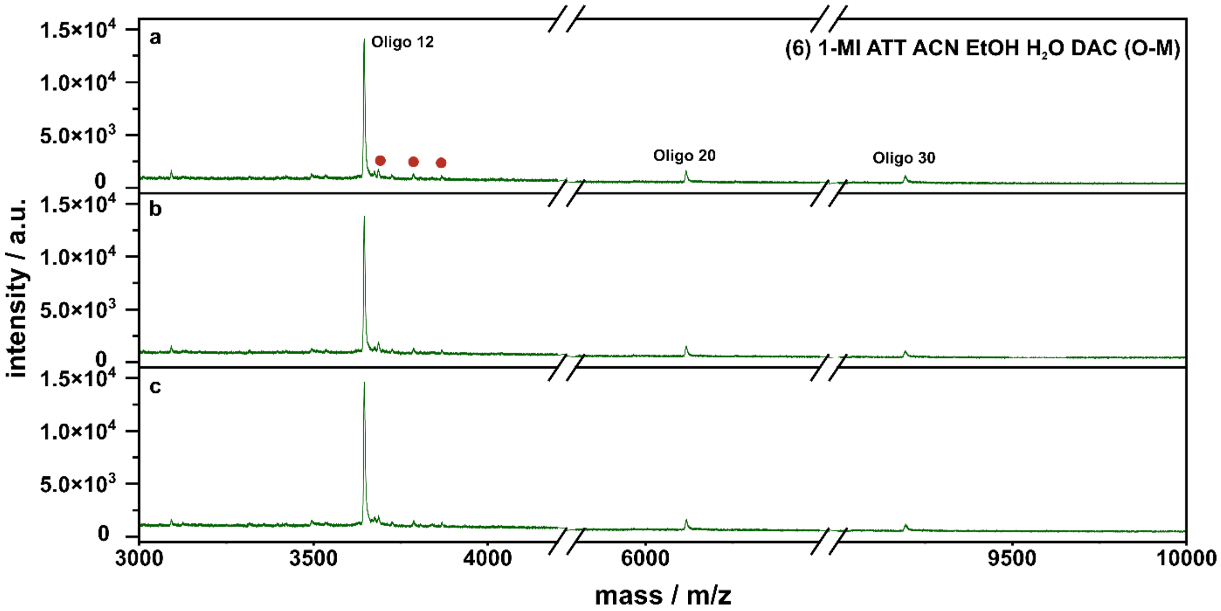

Figure S1: *Mass spectra of three technical replicates (a, b, c) of the MALDI Matrix 1-Methylimidazole (1-MI) with 6-aza-2-thiothymine (ATT) in ACN, EtOH, H_2O, and DAC (sample 6). The oligo-standard (Oligo 12, 20, 30) was spotted prior to the Matrix (O-M). Adducts are highlighted with a red dot.*

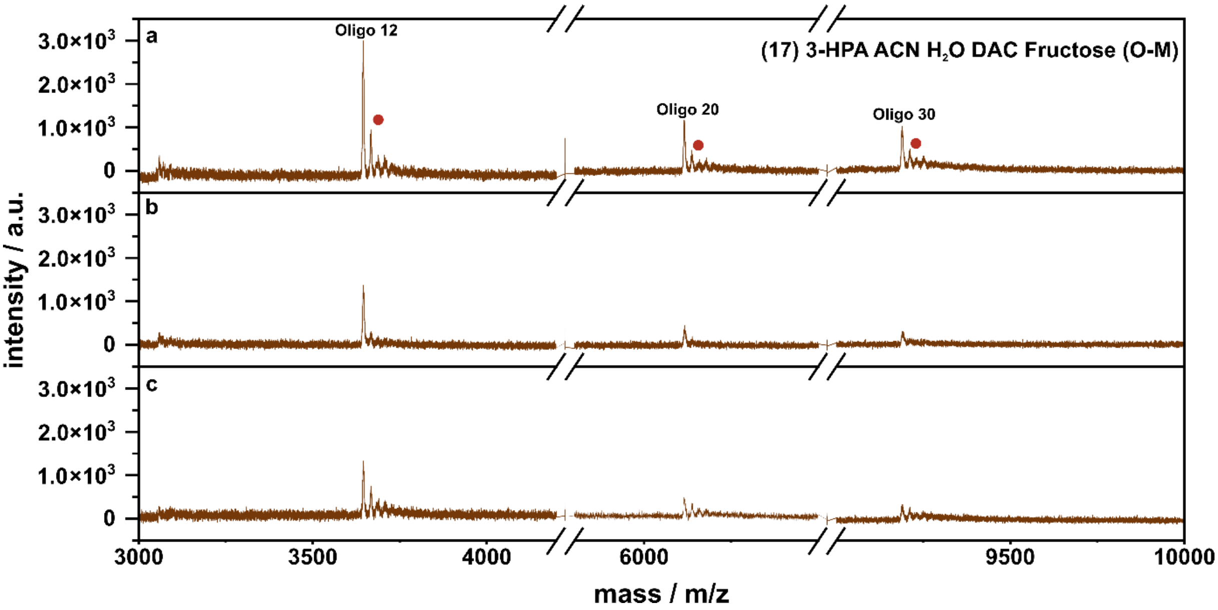

Figure S2: *Mass spectra of three technical replicates (a, b, c) of the MALDI Matrix in ACN, H_2O, DAC, and Fructose (sample 17). The oligo-standard (Oligo 12, 20, 30) was spotted prior to the Matrix (O-M). Adducts are highlighted with a red dot.*
